# Reducibility as a prognosis factor of progression in idiopathic scoliosis

**DOI:** 10.1186/1748-7161-7-S1-P19

**Published:** 2012-01-27

**Authors:** Blanca Palomino, Alicia Villareal, Lorenzo Jiménez, Avelino Ferrero, José Acosta

**Affiliations:** 1 Hospital Ramón y Cajal, Madrid, Spain

## Background

Scoliosis is a disease that develops during growth and knowledge of scalability is essential for making treatment decisions. Of the many factors analyzed, are evidence of sex, topography, menarche , age, rapid growth spurt the magnitude of the curve, Risser 0 - 1 and the imbalance of occipital axis> 10 mm. The degree of reductibility or flexibility is also seen as a predictor of progression during grotw. In this line we have wanted to study whether there is correlation between the initial reduction with cast plaster and outcome in the medium and long term.

## Matherials and methods

This is a retrospective study of 50 patients in the service of Physical Medicine and Rehabilitation of our hospital, which have required orthopedic reduction with a cast and subsequent follow-upcorset. Were analyzed for age, sex, topography of the curve, initial angle value, correction after the cast, final angular value, duration.

## Results

Of the initial sample in 35° of cases whose initial correction with the cast was less than 10% had a progression more than 6° at final follow up (8 years), and 65% of the sample progression did not exceed initial angular value on more than 6°. Cases where the cast correction exceeded 10% at follow-up (8 years), 6% exceeded 6, and 94% remained stable compared to the initial angular value, not to exceed 6 °.

## Conclusions

The initial reducibility factor by treating orthopedic cast, you can be aware of how predictive of long-term outcome.

